# Design and Fabrication of Artificial Stem Cell Niches

**DOI:** 10.3390/bioengineering9120813

**Published:** 2022-12-16

**Authors:** Tiago G. Fernandes

**Affiliations:** 1 Department of Bioengineering and Institute for Bioengineering and Biosciences, Instituto Superior Técnico, Universidade de Lisboa, Av. Rovisco Pais, 1049-001 Lisbon, Portugal; tfernandes@tecnico.ulisboa.pt; 2 Associate Laboratory i4HB—Institute for Health and Bioeconomy, Instituto Superior Técnico, Universidade de Lisboa, Av. Rovisco Pais, 1049-001 Lisbon, Portugal

The term “cellular microenvironment” is a generic expression used to describe the complex collection of stimuli that contribute to cell and tissue functions [1]. This “niche” refers to specific anatomic locations that regulate how cells behave during tissue homeostasis or during dynamic processes, such as tissue development, tissue repair, and malignant transformation [2]. Several factors can be recognized within this niche, particularly soluble molecules, such as hormones, growth factors, cytokines and chemokines that influence cell activities in an autocrine, paracrine, or endocrine fashion. Other stimuli, more physical in nature, include cell–cell interactions and interactions between cells and components of the extracellular matrix. Additionally, the topological features of the niche, its three-dimensionality, and even the stiffness of its native environment can influence cell behavior. Finally, oxygen tension and other physicochemical elements of the environment, including pH, ionic strength (e.g., Ca^2+^ levels) and metabolites, such as ATP, are also significant. Both cells and the niche may stimulate and mutually signal each other to maintain functions and regulate responses during development and adulthood.

Moreover, the native cellular niche plays a critical role in dictating, for example, the fate of stem cells [3]. Therefore, the capacity to recapitulate specific features of such intricate environments can open the door to the development of more efficient biomaterials for tissue regeneration [4]. In fact, due to their characteristics, stem cells from different sources are now considered promising candidates for cell replacement therapies, as well as for drug screening applications and developmental biology studies [5]. This promise, however, has been hampered by the lack of control over the cell fate, since culture conditions capable of directing their differentiation into specific cell types are not well-known. This is mostly due to two reasons. On the one hand, the knowledge of the endogenous or native stem cell niche is incomplete [6]. On the other hand, the capacity to recreate this niche in vitro is very limited [7]. Thus, an increased understanding of stem cell regulatory mechanisms in their endogenous niches may become fundamental to the development of stem-cell-based therapies in the future. Due to the high relevance and complexity of this issue, this niche is presently one of the most important and popular areas of research in the field of stem cells [8].

Many research groups are now directing their efforts towards the design and manufacture of topographically controlled biomaterials that are capable of mimicking different aspects of the native stem cell niche [9]. This is typically achieved by studying both the physical and spatial distribution of the niche, as well as its biochemical and biomechanical properties [10]. This Special Issue therefore focuses on the “Design and Fabrication of Artificial Stem Cell Niches” and aims to communicate innovative methodologies and advancements in the designing of functional and synthetic niche environments.

The latest advancements in bioengineering have allowed for the control of individual aspects of the cellular microenvironment. Therefore, this Special Issue focuses on collecting outstanding contributions covering recent approaches used to recreate and promote the formation of tissue-like structures from cells and materials.

Abdul-Al et al. begin by discussing the importance of stem cell niches in the human eye [11]. They review recent progress in the efforts to recreate the human cornea and the ocular system in vitro. Firstly, they describe the different components of the cornea, which include a pool of self-regenerating epithelial cells that are crucial to preserving clarity and visibility. In particular, the limbal stem cell niche, located at the boundary of the cornea and the conjunctiva, is described. This niche generates a microenvironment that supports the growth and repair of the tissue. Dysfunction affecting any part of the limbal niche can cause disorders and vision impairment. Therefore, the authors describe a series of methods that have been used for regenerating the limbal niche. Among these methods are the use of scaffolds/matrices, mesenchymal stem cells and hemoderivatives. Finally, the authors mention the potential of molecular techniques to classify cells and investigate the relationships between cells and the microenvironment in a precise and systematic manner. On a similar note, Hidalgo-Alvarez et al. discuss the progress made in the replication of stem cell niches from the anterior ocular segment using bioengineering approaches and their therapeutic implications [12]. These authors present biofabrication methods employed for the generation of these artificial cellular microenvironments, including photolithography, electrospinning, and 3D bioprinting. The implications of the development of biomimetic niches in the progress of ocular stem cell therapies are comprehensively discussed.

Therefore, given the importance of microfabrication in the generation of artificial cellular niches, Ramos-Rodriguez et al. present microfabrication techniques used in the design and manufacture of cell microenvironments for tissue regeneration [13]. In fact, several approaches have been employed to recreate the biological components of the native cell niche. The authors focus on describing advanced photolithographic techniques, patterned hydrogels, microfluidic devices, electrospinning-based methods, and other relevant systems, including bioprinting and combination approaches used to replicate specific aspects of the stem cell microenvironment. This research group previously demonstrated that, by combining electrospun fibers and additive manufacturing, it is possible to replicate certain aspects of the skin microenvironment. Furthermore, they explored the use of novel proangiogenic compounds to improve the vascularization of skin constructs. In this Special Issue, they combine both approaches to fabricate innovative polycaprolactone scaffolds loaded with bioactive compounds for skin regeneration and ultimately wound healing [14]. On a different note, Liu et al. used the decellularization and lyophilization of lung tissue to improve the biocompatibility and epithelialization of synthetic tracheal grafts [15].

In summary, different fabrication techniques were developed for the successful generation of artificial microenvironments. Future tissue engineering scaffolds will also benefit from the identification of the critical elements of a given cell niche since this will influence the complexity and functionality of the construct. This capacity to produce dynamic 3D environments where stem cells are capable of residing and differentiating is particularly important for the recreation of bone marrow and the hematopoietic niche [16].

In conclusion, this Special Issue, entitled “Design and Fabrication of Artificial Stem Cell Microenvironments”, offers to the readers of *Bioengineering* the essential topics and exciting future innovations related to the research in this area. It is now evident that many research efforts are being directed towards the design and manufacture of topographically controlled biomaterials that can mimic different aspects of the native stem cell niche. Furthermore, this Special Issue provides outstanding insights into relevant issues related to stem cell research and regenerative medicine.
